# HER2-Low Gastric and Gastroesophageal Junction Adenocarcinoma: From Assessment to Treatment Strategies

**DOI:** 10.3390/ijms27114673

**Published:** 2026-05-22

**Authors:** Alexandra Georgiana Scurtu, Daniela Tatiana Sala, Ioan Jung, Tivadar Bara, Radu Mircea Neagoe, Zsolt Zoltán Fülöp, Simona Gurzu

**Affiliations:** 1 Department of Pathology, George Emil Palade University of Medicine, Pharmacy, Science and Technology, 540139 Targu Mures, Romania; alexandra.dr03@yahoo.com (A.G.S.); jungjanos@studium.ro (I.J.); 2 2nd Clinic of Surgery, Mureș County Emergency Clinical Hospital, 540136 Targu Mures, Romania; btibi_ms@yahoo.com (T.B.); neagoerm@gmail.com (R.M.N.); 3 Doctoral School of Medicine and Pharmacy, George Emil Palade University of Medicine, Pharmacy, Science and Technology, 540139 Targu Mures, Romania; 4 Department of Surgery, George Emil Palade University of Medicine, Pharmacy, Science and Technology, 540139 Targu Mures, Romania; 5 Romanian Academy of Medical Sciences, 030167 Bucuresti, Romania; 6 Szegedi Tudományegyetem, Szent-Györgyi Albert Klinikai Központ, Sebészeti Klinika, 6725 Szeged, Hungary; zsolt_fulop15@yahoo.com; 7 Research Center of Oncopathology and Translational Medicine (CCOMT), 540136 Targu Mures, Romania

**Keywords:** gastric cancer, gastroesophageal junction adenocarcinoma, HER2-low, antibody–drug conjugate, trastuzumab deruxtecan, disitamab vedotin

## Abstract

Human epidermal growth factor receptor 2 (HER2) dysregulation contributes to tumorigenesis in gastric and gastroesophageal junction adenocarcinomas (GC/GEJ). HER2 overexpression has been associated in multiple cohorts with aggressive behavior and poor outcomes. While HER2 amplification has long guided therapy in HER2-positive disease, antibody–drug conjugates (ADCs) have shifted attention toward the HER2-low category, typically defined as immunohistochemistry (IHC) 1+ or IHC 2+ with negative in situ hybridization (ISH). This narrative review integrates evidence from the peer-reviewed literature, current testing recommendations, and registered clinical trials. It clarifies practical issues in HER2-low assessment and maps the evolving therapeutic landscape of HER2-targeted ADCs including rational combination strategies that may extend benefit beyond conventionally HER2-positive tumors. A cross-tumor perspective contrasts GC/GEJ testing and biology with the breast cancer paradigm and summarizes the importance of HER2-low expression in non-gastric malignancies. Finally, we discuss the therapeutic strategies in HER2-low GC/GEJ and highlight key safety and monitoring considerations for HER2-directed ADCs.

## 1. Introduction

Gastric cancer (GC) is a heterogeneous, multifactorial malignancy in which *Helicobacter pylori* (*H. pylori*—class I carcinogen) is the dominant established risk factor. This association has been recognized for decades, since the International Agency for Research on Cancer classified *H. pylori* as a Group 1 definite carcinogen in 1994, and earlier studies also emphasized its role in chronic gastritis, peptic ulcer disease, GC risk, and eradication-based prevention strategies [1]. Earlier clinical studies also highlighted the relevance of *H. pylori* detection and antimicrobial susceptibility testing in gastric biopsy specimens, reflecting its broader role in gastric disease management [2]. Epstein–Barr virus (EBV) infection is also implicated in gastric carcinogenesis and defines a distinct molecular subtype of GC [3]. In rare but well-documented cases, familial aggregation and hereditary predisposition may occur, particularly in hereditary diffuse gastric cancer and selected familial cancer syndromes [4]. Etiology varies by tumor location: chronic *H. pylori*-driven inflammation is more typical of distal and mid-gastric tumors, whereas obesity and gastroesophageal reflux are more associated with proximal GC or gastroesophageal junction (GEJ) adenocarcinomas [5].

Despite declining incidence in some regions, GC remains a major global health burden, with nearly one million new cases reported annually [6]. The close alignment between incidence and mortality reflects poor survival driven by late-stage diagnosis and limited efficacy of therapies in metastatic disease, underscoring the need for effective screening and prognostic biomarkers to reduce mortality and increase therapeutic success.

Early symptoms of GC/GEJ adenocarcinoma are frequently absent or non-specific, and patients may present with weight loss, anorexia, epigastric discomfort, anemia, or other features that prompt diagnostic evaluation. Upper gastrointestinal endoscopy with biopsy remains central to confirming the diagnosis and provides the tissue basis for biomarker testing. In this context, HER2 and microsatellite instability/mismatch repair (MSI/MMR) status have been mentioned as relevant biomarkers, while programmed death-ligand 1 (PD-L1) expression and claudin 18.2 (CLDN18.2) are increasingly incorporated into the therapeutic biomarker framework of advanced GC/GEJ adenocarcinoma [7,8].

Historically, HER2 has been approached as a binary biomarker, primarily to select patients for conventional anti-HER2 therapies in tumors with HER2 overexpression or gene amplification.

With the advent of antibody–drug conjugates (ADCs), low-level HER2 expression has gained therapeutic relevance (Table 1), expanding interest beyond the traditional HER2-positive population [9,10].

This review aims to consolidate and critically evaluate the evidence regarding HER2-low expression in GC and GEJ adenocarcinomas. Specifically, it addresses the biological basis of HER2 signaling, the epidemiology and prognostic implications of HER2-low tumors, and the current therapeutic strategies under investigation, particularly ADCs. Additionally, comparisons with HER2-low expression in other malignancies, such as breast, lung, and pancreatic cancer, are explored. We aimed to present an evidence-based synthesis that may help clinical decision-making and highlight key areas for future research in HER2-low GC.

A targeted literature search was conducted in PubMed, Scopus, and Web of Science (primarily 2015–2026; earlier sources for definitions when needed) using terms related to HER2-low expression in GC/GEJ adenocarcinomas and ADCs. ClinicalTrials.gov was additionally screened to capture ongoing and completed programs relevant to HER2-low/HER2-intermediate populations, and key conference abstracts were considered when full publications were unavailable. Eligible sources included peer-reviewed studies, guidelines, registered clinical trials, and relevant conference abstracts addressing HER2 assessment or HER2-directed treatment in GC/GEJ adenocarcinoma. Sources were excluded if they did not include GC/GEJ adenocarcinoma, did not report HER2 status or HER2-related eligibility criteria, or lacked sufficient information on study design or treatment strategy. Clinical trials were selected, as shown in Table 2, when they included a dedicated HER2-low or HER2-intermediate/low cohort, subgroup, or exploratory analysis, regardless of treatment regimen. Evidence was synthesized qualitatively for this narrative review, without formal risk-of-bias assessment or meta-analysis.

## 2. Overview

### 2.1. Molecular Classification of GC and Implications for GC/GEJ Biology

Traditional histologic systems used for classification of GC—such as the Lauren classification (intestinal vs. diffuse) and the World Health Organization (WHO) categories (papillary, tubular, mucinous [colloid] and poorly cohesive carcinomas)—capture morphologic heterogeneity but offer limited direct utility for treatment selection [11,12]. Other studies have profiled GC at the genomic, epigenomic, transcriptomic, proteomic, and metabolomic levels, revealing recurrent alterations and pathway dysregulations with potential prognostic and therapeutic relevance [13]. These datasets have enabled the development of several molecular classification frameworks, most notably those proposed by The Cancer Genome Atlas (TCGA) and the Asian Cancer Research Group (ACRG). However, their routine clinical impact remains limited, largely because implementation is complex and requires resources not widely available.

In 2014, TCGA analyzed 295 primary gastric adenocarcinomas using integrated genomic and molecular profiling and proposed a new molecular classification with four groups: EBV-positive, microsatellite instability (MSI), genomically stable (GS), and chromosomal instability (CIN) [14].

The Asian Cancer Research Group performed integrated molecular profiling of 251 GCs (gene expression, copy-number analysis, and targeted sequencing) and proposed a four-subtype classification with clear prognostic stratification [15]. ACRG defined the following categories: MSI, microsatellite stable with epithelial–mesenchymal transition (MSS/EMT), MSS/TP53 aberrant/active (MSS/TP53+), and MSS/TP53 inactive (MSS/TP53−) [15,16]. Clinically, MSI tumors are predominantly intestinal-type, often antral, and associate more favorable outcomes among ACRG subtypes [15,17,18]. In contrast, the MSS/EMT subtype was enriched for diffuse histology, tended to present at a younger age, and showed the highest recurrence risk [15,19]. The remaining MSS/TP53+ and MSS/TP53− subgroups exhibit intermediate outcomes, with comparatively better prognosis in MSS/TP53+ than in MSS/TP53− tumors [13,15].

While TCGA/ACRG subtypes do not map directly onto routine clinical workflows, they remain useful for treatment-oriented strategies. In this context, HER2 assessment is particularly challenging because expression can be spatially heterogeneous and often lies near low-expression thresholds, making accurate testing and reporting critical as HER2-low emerges as a treatment-relevant category. Although TCGA/ACRG molecular classifications were developed primarily for gastric adenocarcinoma, GC frameworks are used here as a practical reference when discussing GC/GEJ biology, recognizing that site-related differences may exist.

### 2.2. HER2 Biology and Role in Carcinogenesis and Targeted Therapy

HER2, an oncogenic driver, is a transmembrane glycoprotein within the epidermal growth factor receptor (EGFR) family. The HER2 gene, located on chromosome 17q21, encodes HER2 protein (also known as ErbB-2) and is both a key driver in carcinogenesis and a critical therapeutic target, especially in the treatment of breast cancer and GC [20]. Aberrant HER2 signaling is implicated in the pathogenesis of a wide range of malignancies, and its amplification/overexpression is associated with a worse clinical outcome [21]. While initially observed and studied in breast cancer, HER2 dysregulation and ERBB2 alterations have also been described across multiple solid tumors, including GC, non-small cell lung cancer (NSCLC), bile duct cancer, urothelial carcinoma, and colorectal cancer [22,23,24,25]. HER2 amplification or protein overexpression has been successfully targeted in breast cancer and subsequently in GC, particularly following the phase III clinical study ToGA, which demonstrated an improved survival with trastuzumab in combination with chemotherapy in HER2-positive advanced GC [25].

HER2-targeted drugs, such as specific monoclonal antibodies (trastuzumab), tyrosine kinase inhibitors (TKIs) like neratinib, and ADCs like trastuzumab deruxtecan (T-DXd), have been approved by the U.S. Food and Drug Administration and effectively inhibit HER2 pathways [21]. Advances in molecular research continue to drive the development of these targeted strategies, with ADCs notably transforming treatment response across several HER2-positive cancers [26].

Recent molecular insights into GC have emphasized HER2 as a key biomarker with important prognostic and predictive value.

### 2.3. HER2 Assessment in Gastric and GEJ Adenocarcinoma

#### 2.3.1. Testing Methodology

**Figure 1:**
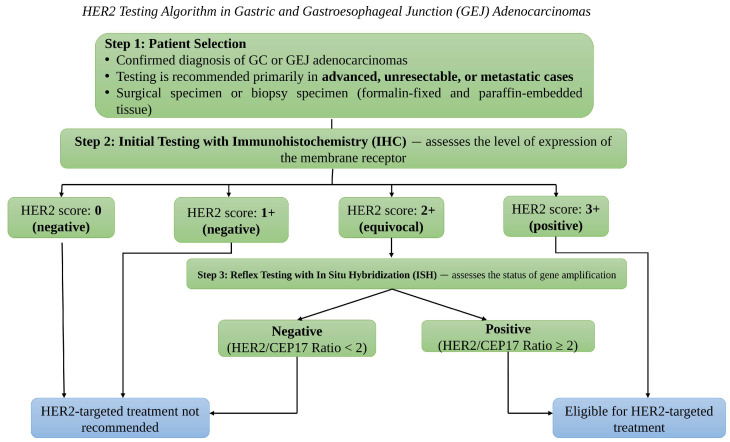
HER2 testing algorithm in GC/GEJ adenocarcinomas. Stepwise diagnostic workflow based on initial IHC scoring with reflex in situ hybridization (ISH) when indicated to confirm HER2 status for treatment selection. Created by the authors; adapted from guideline-based diagnostic workflows [29,30].

Given that HER2 testing and treatment evidence is commonly reported for GC/GEJ adenocarcinomas together, we use the term GC/GEJ in the HER2-focused sections of this review. Immunohistochemistry (IHC) remains the primary method for detecting HER2 protein overexpression in tumor tissues, with results categorized as positive (3+), equivocal (2+), or negative (zero or 1+) based on standardized scoring criteria. Complementary techniques, such as fluorescence in situ hybridization (FISH) and chromogenic in situ hybridization (CISH), are critical for confirming HER2 gene amplification. HER2-targeted therapies, including monoclonal antibodies and tyrosine kinase inhibitors, have demonstrated efficacy as neoadjuvant/adjuvant treatments and as systemic therapies for unresectable or metastatic disease [27]. Current international guidelines (e.g., ESMO, CAP/ASCO) and empirical evidence consistently recommend analyzing multiple (5–8) biopsy fragments or tissue blocks per case. This practice addresses intratumoral HER2 heterogeneity, a well-recognized phenomenon in GC, improves detection accuracy, and reduces false negatives—highlighting that HER2 evaluation remains a persistent diagnostic challenge in GC [28,29]. The HER2 testing workflow in GC/GEJ adenocarcinomas is summarized in Figure 1.

#### 2.3.2. Pattern of Expression and Heterogeneity

GC/GEJ adenocarcinomas exhibit distinct HER2 immunostaining features compared with breast cancer, most notably with incomplete basolateral/lateral (“U-shaped”) membranous staining and a high prevalence of focal/patchy expression; therefore, IHC requires gastric-specific interpretation [31,32,33,34]. Within these criteria (as implemented in ToGA), strong incomplete membrane staining is considered HER2-positive when present in ≥10% of tumor cells in resections or in a cluster of ≥5 tumor cells in biopsies, highlighting why adequate multi-site sampling is critical when only limited biopsy tissue is available [25,33,35]. For a reliable assessment of HER2 status, it is recommended to examine at least 3 to 4 histological slides. In addition, FISH should be performed in any case presenting focal clusters of HER2-positive immunoexpression, even when such clusters comprise less than 5% of tumor cells [36]. Clinically, intratumoral heterogeneity—with coexistence of HER2-positive and HER2-negative areas—can be observed in a substantial subset of cases (reported up to ~26% in metastatic/unresectable cohorts) and has been associated with worse overall survival (OS) [37]. Discordance between IHC and in situ hybridization (ISH), including FISH, in GC/GEJ adenocarcinomas has been attributed to the characteristic incomplete basolateral/lateral membranous staining pattern of gland-forming tumor cells together with intratumoral heterogeneity, reinforcing the need for careful interpretation—particularly in limited biopsy material [32,33].

#### 2.3.3. HER2-Low Concept

HER2 status is conventionally classified, after IHC evaluation, as HER2-positive (IHC 3+ or IHC 2+ with ISH positivity/amplification) or HER2-negative (IHC 0/1+, or IHC 2+ with ISH-negative results) [9]. Notably, IHC 2+ represents an equivocal category by IHC alone and requires confirmatory ISH to assign final HER2 status (Figure 1) [38]. However, many GCs exhibit low but detectable HER2 expression—defined as HER2-low (typically IHC 1+ or IHC 2+/ISH-negative)—a subgroup previously managed within the broader HER2-negative category [39].

The concept of HER2-low expression has attracted increasing attention due to its potential therapeutic implications. In a retrospective cohort study involving 548 patients with advanced GCs, 45.1% of tumors were classified as HER2-low, compared to 33.0% HER2-zero (IHC 0) and 21.8% HER2-positive cases (IHC 3+ or IHC 2+/ISH+) [40]. Another larger retrospective cohort study including 994 patients reported that HER2-low GC accounted for 24.9% of cases and was associated with distinct clinicopathological features compared with HER2-zero disease, including older age, ethnicity-related differences, cardia/fundus predominance, adenocarcinoma histology, less advanced T stage, and a higher proportion of metastatic disease. HER2-low status was also associated with worse OS in the overall cohort and emerged as an independent adverse prognostic factor in non-metastatic disease [41]. In early-stage GC, HER2-low status was observed in 31.8% of cases in a study analyzing 157 patients [42], supporting the view that HER2-low GC may represent more than a purely descriptive subgroup within HER2-negative tumors [41].

This interest is amplified by the current limitations of HER2-targeted therapy in HER2-positive GC/GEJ adenocarcinomas. Although trastuzumab-based approaches have demonstrated clinical efficacy, long-term benefit is often limited, and many patients ultimately experience disease progression due to therapeutic resistance. This resistance is primarily driven by intratumoral heterogeneity of HER2 expression, loss of HER2 gene amplification during treatment, and activation of alternative oncogenic signaling pathways. These challenges highlight the need for more durable and personalized treatment strategies in HER2-positive GC [43,44]. Against this background, the evolving HER2-low paradigm—initially established as clinically actionable in breast cancer—has reshaped therapeutic thinking by emphasizing that low-level HER2 expression may still be therapeutically exploitable, even when tumors do not meet traditional HER2-positive criteria. Inspired by these advances, increasing attention is now being directed toward defining the significance of HER2-low expression in GC.

#### 2.3.4. Practical Gray Zones in HER2-Low Classification

Beyond the formal definition of HER2-low, real-world implementation in GC/GEJ adenocarcinoma is limited by several practical gray zones. Available data specifically addressing IHC 0 versus IHC 1+ reproducibility in GC/GEJ adenocarcinomas remain limited, because traditional gastric HER2 testing was designed primarily to identify HER2-positive disease rather than to separate HER2-zero from HER2-low tumors. In the gastric-specific validation study by Rüschoff et al., interrater agreement was lower when all IHC intensity categories were considered separately, whereas concordance improved when scores were grouped as negative, IHC 0/1+, versus positive/equivocal, IHC 2+/3+ [31]. More directly, in HER2-low gastroesophageal adenocarcinoma, the IHC 0 versus IHC 1+ boundary has been identified as a key reproducibility challenge, with previous studies reporting the lowest agreement between HER2 0 and HER2 1+ cases and real-world data showing reduced interobserver and interlaboratory concordance, especially in biopsy specimens [45].

Focal HER2 expression represents another important source of discordance in GC/GEJ adenocarcinoma. Gastric-specific HER2 scoring systems recognize this issue by applying different criteria to biopsy and resection material: in biopsies, HER2 positivity requires at least five cohesive, unequivocally stained tumor cells, whereas the 10% cut-off remains applicable to resection specimens [31,33]. With the development of HER2-directed ADCs, focal or low-level HER2 expression may be relevant for clinical-trial eligibility. However, the minimum amount, intensity, and spatial distribution of HER2 expression required for ADC benefit in GC/GEJ adenocarcinoma remain undefined. Therefore, focal HER2-low staining should prompt careful pathological review and, where appropriate, additional sampling or reflex ISH, but should not be considered independently clinically actionable outside trial-defined criteria or approved indications [29,45].

The distinction between HER2-low, HER2-ultralow, and HER2-zero also remains unsettled in GC/GEJ adenocarcinoma. HER2-ultralow has mainly been developed in breast cancer to describe extremely faint or barely perceptible incomplete membranous staining within the IHC 0 range, whereas HER2-null indicates complete absence of staining [46]. Although this terminology has growing therapeutic relevance in breast cancer, it has not yet been standardized or prospectively validated in GC/GEJ adenocarcinoma, and extrapolation to routine GC decision-making should therefore be avoided outside clinical trials.

Specimen selection further complicates HER2-low assessment. HER2-low expression may evolve during metastatic progression, and primary-tumor HER2 status does not always reliably predict HER2 status in metastatic lesions. In a paired primary–metastatic GC study evaluating HER2-low evolution from primary to distant metastatic lesions, He et al. reported an overall HER2 discordance rate of 48.8%. Notably, 32.7% of patients with HER2-negative primary tumors developed HER2-low metastatic disease [47]. Earlier paired analyses also support assessment beyond the primary tumor. Fusco et al. found discordant HER2 status between primary GC and synchronous lymph-node metastases in 14% of cases, including HER2-positive lymph-node metastases arising from HER2-negative primary tumors, highlighting the therapeutic relevance of assessing both primary and metastatic sites when feasible [48]. Therefore, when feasible, HER2-low assessment should use the most clinically relevant and recent available tumor tissue, and repeat testing of metastatic or recurrent lesions may be considered, especially when the original sample was limited, heterogeneous, or obtained before systemic therapy [29,47].

## 3. HER2-Low as a Therapeutic Concept Across Non-Gastric Malignancies

### 3.1. Breast Cancer as Proof of Concept for HER2-Low Targeting

The pivotal proof of concept for HER2-low targeting emerged with the anti-HER2 ADC T-DXd in DESTINY-Breast04, a phase III trial enrolling patients with previously treated HER2-low metastatic breast cancer. Among all patients, median PFS was 9.9 vs. 5.1 months and median OS was 23.4 vs. 16.8 months, establishing HER2-low as a therapeutically actionable category. Interstitial lung disease (ILD)/pneumonitis remained a key safety consideration requiring vigilant monitoring [10].

In DESTINY-Breast06 (HR-positive, HER2-low metastatic disease after endocrine-based therapy), T-DXd prolonged PFS versus physician’s choice (PC) chemotherapy (13.2 vs. 8.1 months; HR 0.62), while OS data were immature and adjudicated ILD/pneumonitis occurred in 11.3% (including fatal events) [49].

In the phase II DAISY trial, T-DXd demonstrated clinically meaningful efficacy in metastatic HER2-low breast cancer, with a confirmed objective response rate of 37.5% (95% CI, 26.4–49.7), meeting the study’s primary endpoint for this cohort. Responses were also observed in an IHC 0 cohort (confirmed ORR 29.7%, 95% CI, 15.9–47.0), supporting the concept of a continuum of breast cancer sensitivity across low and absent HER2 expression. No new safety signals were reported [50].

With proof of concept established in breast cancer, the next question is whether comparable ADC-driven benefits can be reproduced in other solid tumors characterized by heterogeneous and often low-level HER2 expression.

### 3.2. Urothelial Carcinoma

HER2 is increasingly recognized as a therapeutically relevant target in metastatic urothelial carcinoma (UC). In a phase II study of disitamab vedotin (DV; also known as RC48), previously treated metastatic UC patients with study-defined HER2-negative/low disease (IHC 0 or 1+) were evaluated. The confirmed ORR was 31.6% (95% CI, 12.6–56.6) and the disease control rate was 94.7% (18/19). Median PFS was 5.5 months and median OS was 16.4 months, supporting the potential clinical activity of HER2-directed ADC therapy even at low levels of HER2 expression in this setting [51].

### 3.3. Endometrial/Uterine Malignancies

In gynecologic malignancies, uterine carcinosarcoma (UCS) provides one of the clearest signals that low-level HER2 expression may be clinically meaningful in the context of HER2-directed ADC development. In the phase II STATICE trial of T-DXd in recurrent UCS with HER2 IHC ≥ 1+, responses were observed in both study-defined HER2-high (IHC ≥ 2+) and HER2-low (IHC 1+) cohorts. ORR was 54.5% vs. 70.0% and median PFS was 6.2 vs. 6.7 months. ILD/pneumonitis was reported in 27% of patients overall (grade 1–2: 24%; grade 3: 3%) [52]. In a retrospective UCS cohort scored using gastric ASCO/CAP criteria, HER2-low (defined in that study as IHC 1+) was common (38.5%) and showed low IHC–FISH concordance (FISH positivity 4.8% in IHC 1+). In stage I/II disease, IHC 0 and IHC 1+ cases had worse OS than HER2-high tumors (median OS 40 and 31 vs. 57 months; *p* = 0.033), supporting IHC-based stratification and highlighting an unmet need for improved systemic strategies [53].

### 3.4. Ovarian Cancer

In ovarian cancer, evidence supporting HER2-low as a distinct therapeutic category remains limited, with data largely confined to case reports and ongoing trials. A reported ovarian carcinosarcoma case with HER2 IHC 1+ and FISH-negative status achieved a durable partial response to T-DXd, suggesting potential activity in selected low-expressing tumors [54]. Building on this rationale, the ongoing phase III DESTINY-Ovarian01 trial (NCT06819007) is evaluating T-DXd plus bevacizumab versus bevacizumab monotherapy as first-line maintenance therapies in patients with HER2-expressing ovarian cancer, including IHC 1+ disease, according to registry information [55].

### 3.5. Lung Cancer

In non-small cell lung cancer, the clinical relevance of the breast-derived HER2-low category remains uncertain, as there is currently no prospective evidence demonstrating that IHC 1+ reliably predicts benefit from HER2-directed ADCs. Instead, the strongest clinical validation of HER2-targeted therapy in lung cancer comes from ERBB2 (HER2) activating mutations treated with T-DXd, where substantial and durable responses have been reported, establishing HER2 as an actionable driver alteration in this setting [56,57]. In HER2-overexpressing NSCLC, ADC activity has primarily been evaluated using IHC 2+/3+ selection thresholds. Although responses have been observed, these cohorts do not capture the IHC 1+ (HER2-low) population, highlighting a clear evidence gap for low-expression NSCLC and underscoring the need for dedicated prospective studies [58].

### 3.6. Pancreatic Cancer

In pancreatic ductal adenocarcinoma (PDAC), HER2-low appears biologically and clinically relevant, although its predictive value for HER2-directed therapy remains uncertain. Han et al. reported HER2-low PDAC (IHC 1+ or IHC 2+/ISH-negative) in 18/55 patients (32.8%), which was associated with poorer outcomes, with additional adverse impact of HER2 genetic heterogeneity, supporting the rationale for next-generation anti-HER2 approaches in selected subsets [59]. Prospective evidence for T-DXd in PDAC remains limited, as in DESTINY-PanTumor02, activity in the pancreatic cohort was low, with discordant response estimates between investigator assessment and central review, suggesting at most a modest signal in a small subgroup and underscoring challenges in patient selection [60]. In a DESTINY-PanTumor02 subgroup analysis reported as a conference abstract, central reassessment showed clinically relevant discordance between local and central HER2 testing in PDAC. Some locally HER2-expressing tumors were reclassified as IHC 1+ or IHC 0, highlighting analytical and interpretive limitations—especially at the low end of HER2 expression PDAC [61].

## 4. Therapeutic Options in HER2-Low Gastric and GEJ Adenocarcinoma

Across the evidence reviewed, HER2-low in GC/GEJ adenocarcinomas is best framed as a clinically relevant eligibility window created by the ADC era rather than a newly delineated biological subtype. Its relevance derives from the possibility that HER2-directed ADCs may retain activity in tumors with low or heterogeneous HER2 expression, although dedicated outcome reporting is still needed [10,39,62].

Beyond conventional IHC/ISH categories, several candidate refinements may better characterize tumors currently grouped as HER2-low. Digital or quantitative IHC may improve the reproducibility of borderline HER2 scoring. For example, HER2-CONNECT reduced equivocal classifications in GC/GEJ adenocarcinoma after assay-specific cut-off adjustment, although further validation is required [63]. Molecular assays may provide additional information. ERBB2 mRNA expression has recently been explored in advanced GC as a quantitative biomarker beyond standard HER2 pathology [64]. Next-generation sequencing (NGS)-based ERBB2 copy-number assessment may capture gene dosage alterations that are not fully reflected by binary amplified versus non-amplified categories [65]. Spatial heterogeneity measures could complement HER2-low classification by quantifying the distribution of HER2 expression within the primary tumor and across metastatic sites. These measures may include the proportion and spatial distribution of HER2-expressing tumor areas, focal HER2-positive clusters, and primary–metastatic discordance [66,67]. Their clinical utility in HER2-low GC/GEJ has not yet been established.

Conventional, unconjugated HER2 blockade with trastuzumab is not supported for HER2-low GC/GEJ adenocarcinomas and is recommended only for HER2-positive disease. Notably, in ToGA exploratory analyses, no benefit was observed in tumors with very low/absent HER2 protein expression (IHC 0/1+), even when ERBB2 amplification was present. Benefit was concentrated in high-expression disease. HER2-directed ADCs provide a stronger mechanistic rationale through payload-driven cytotoxicity and potential bystander killing, which may mitigate the limitations of low and heterogeneous antigen density [25,68]. Consequently, therapeutic development in HER2-low GC/GEJ adenocarcinoma currently focuses on HER2-directed ADCs, including monotherapy and combinations with PD-1/PD-L1 checkpoint inhibitors, chemotherapy, or anti-angiogenic agents [62,69]. Because the available evidence ranges from established HER2-positive treatment standards to exploratory and ongoing HER2-low studies, Table 1 summarizes these categories to support clinical interpretation.

### 4.1. ADC Monotherapy (T-DXd and RC48; Emerging ADCs)

Trastuzumab deruxtecan provides the most direct clinical signal in HER2-low GC/GEJ adenocarcinomas based on exploratory cohorts. In the DESTINY-Gastric01 exploratory HER2-low cohorts, T-DXd demonstrated antitumor activity in heavily pretreated patients: confirmed ORR was 26.3% in cohort 1 (IHC 2+/ISH-negative) and 9.5% in cohort 2 (IHC 1+), with disease control rates of 89.5% and 71.4%, respectively. Among responders, the median duration of response was 7.6 months in cohort 1 and 12.5 months in cohort 2, supporting the potential clinical relevance of HER2-directed ADCs even at low HER2 expression levels [70]. However, these exploratory cohorts were not designed to establish HER2-low as a routine treatment-defining biomarker.

Disitamab vedotin is a HER2-directed ADC with established clinical activity primarily in HER2-overexpressing/HER2-positive gastric and GEJ cancer. In a single-arm phase II study of heavily pretreated HER2-overexpressing GC/GEJ adenocarcinomas, DV monotherapy demonstrated promising efficacy with manageable safety, supporting its therapeutic role in HER2-high disease [71]. Early-phase data in advanced HER2-expressing solid tumors further suggested tolerability and activity signals in GC, including patients classified as IHC 2+/ISH-negative in that development program [72]. By contrast, for HER2-low GC/GEJ, the most clinically informative evidence to date comes from DV-based combinations rather than monotherapy. In the phase I RC48-C013 study, DV combined with the PD-1 inhibitor toripalimab produced objective responses in a prespecified HER2-low subgroup, supporting continued evaluation of HER2-ADC plus immunotherapy strategies in HER2-low disease [69].

Beyond these “front-runner” ADCs, several next-generation HER2-ADCs (e.g., SHR-A1811) are in early clinical development in HER2-expressing GC/GEJ adenocarcinomas. Eligibility criteria may include tumors across a range of HER2 expressions (including IHC 1+). However, robust efficacy estimates specifically in HER2-low GC/GEJ adenocarcinomas remain limited and await dedicated reporting [73].

### 4.2. ADC + Immunotherapy

Preclinical evidence supports a biological rationale for combining HER2-directed ADCs with immune checkpoint blockade. In a murine model, T-DXd (DS-8201a) can enhance antitumor immunity and shows improved activity when combined with PD-1 blockade [74]. In the multicenter, open-label phase I RC48-C013 dose escalation/expansion study, DV plus toripalimab (PD-1 inhibitor) was evaluated in pretreated HER2-expressing GC/GEJ adenocarcinomas, including a prespecified HER2-low subgroup. Within the recommended phase II dose (RP2D)-treated GC/GEJ adenocarcinomas cohort, objective responses were observed in both HER2-positive and HER2-low tumors, with ORRs of 56% and 46%, alongside PFS estimates of 7.8 months vs. 5.1 months, supporting further development of HER2-ADC plus PD-1 blockade strategies in HER2-low disease [69]. In a multicenter real-world cohort, adding an immune checkpoint inhibitor (ICI) to RC48 was associated with higher response and disease control rates compared with RC48 alone (ORR 36% vs. 10%, DCR 80% vs. 50%) and a longer median PFS (6.2 vs. 3.9 months) in third-line and later treatments of advanced/metastatic GC. When analyzed by HER2 status, outcomes did not differ significantly between HER2-positive and HER2-low subgroups, suggesting potential activity across the HER2 expression spectrum; however, the non-randomized real-world design limits causal inference [75]. These findings support further investigation but should not be considered definitive evidence for routine HER2-low-directed treatment.

### 4.3. ADC + Anti-Angiogenic and/or Chemotherapy

Combination strategies aim to deepen responses by integrating HER2-directed ADC activity with chemotherapy and/or by modulating the tumor microenvironment and drug delivery through anti-angiogenic agents. In HER2-low GC/GEJ adenocarcinomas, such approaches are being prospectively tested in the first-line setting. Notably, EPOC2203 is an ongoing single-arm phase Ib/II study evaluating T-DXd in combination with nivolumab and reduced-dose capecitabine plus oxaliplatin (CAPOX) as a first-line treatment for HER2-low metastatic GEJ adenocarcinoma. The study was initially reported as a trial-in-progress conference abstract and was subsequently described in a published protocol-style report [76,77]. NCT05980481 is a phase II/III trial evaluating toripalimab + CAPOX with or without RC48 in patients with locally advanced or metastatic GC stratified by trial-defined HER2 expression categories, including a HER2-intermediate/low cohort corresponding to IHC 1+ or IHC 2+/ISH-negative disease. Early conference-style reports are emerging, but final peer-reviewed outcomes for the HER2-low subgroup remain the key benchmark; mature HER2-low-specific efficacy estimates are not yet available [78]. Anti-angiogenic combinations are being explored to potentially enhance intratumoral delivery and modulate immune/stromal barriers. NCT05894824, a phase Ib/II study evaluates T-DXd + ramucirumab specifically in HER2-low unresectable/metastatic GC/GEJ adenocarcinomas [79].

### 4.4. Other HER2-Targeting Formats

Most non-ADC HER2-targeting approaches (unconjugated monoclonal antibodies and many bispecific antibodies) have been developed primarily for HER2-overexpressing or ERBB2-amplified tumors, where higher target density supports receptor blockade and/or immune effector engagement. Accordingly, direct clinical evidence in HER2-low GC/GEJ adenocarcinomas remains limited, and these platforms currently have a less established rationale than HER2-directed ADCs in this subgroup. Bispecific anti-HER2 antibodies such as KN026 have shown clinically meaningful activity in HER2-driven gastric/GEJ adenocarcinoma; however, reported signals appear largely restricted to tumors with ERBB2 amplification/high-level HER2 biology, while responses in tumors with low HER2 expression without amplification have been minimal [80,81]. In the peer-reviewed combined phase Ib/II analysis of KN026 plus KN046, objective responses were observed in HER2-positive non-breast cancers, including HER2-positive GC; in contrast, objective responses were not observed among patients with low HER2 expression included in the early phase Ib component [81]. More broadly, bispecific formats such as zanidatamab are being developed in HER2-positive GC/GEJ adenocarcinoma, but direct evidence in HER2-low disease remains limited [82].

Across contemporary reviews, the prevailing view is that HER2-ADC payload biology currently represents the most actionable axis in HER2-low GC/GEJ, whereas other HER2-targeting platforms remain investigational and will likely require either payload conjugation or refined biomarker strategies to identify HER2-low tumors with HER2-dependent biology [62].

Finally, the HER2-low GC/GEJ adenocarcinomas therapeutic landscape is currently ADC-centric, with (1) T-DXd monotherapy signals in exploratory HER2-low cohorts, (2) RC48 + PD-1 inhibitor showing the most concrete HER2-low combination efficacy to date, and (3) rapidly expanding first-line/combination trials integrating immunotherapy, chemotherapy, and anti-angiogenic agents specifically for HER2-low populations [69,70,76,77,78].

Against this backdrop, the following section summarizes the ongoing and completed clinical trials that are actively shaping HER2-low GC treatment development.

## 5. Ongoing and Completed Clinical Trials in HER2-Low Gastric Cancer

The available trials investigating HER2-low GC are phase I or phase II, predominantly open-label in design, and frequently include HER2-low populations as exploratory subgroups or dedicated cohorts within broader study populations. ADCs targeting HER2, notably T-DXd and RC48, represent the backbone of most of these investigational strategies. Several studies evaluate T-DXd either as monotherapy or in combination with anti-angiogenic agents, cytotoxic chemotherapy, or PD-1 inhibitors, reflecting a growing interest in synergistic treatment approaches (Table 2). Detailed study-level information is available in Supplementary Table S1.

In the pivotal DESTINY-Gastric01 randomized phase II trial, T-DXd demonstrated substantial clinical benefit in patients with previously treated HER2-positive advanced gastric or GEJ adenocarcinoma. In this study, T-DXd achieved an objective response rate of approximately 51% compared with 14% in the PC chemotherapy arm, and median OS was prolonged (12.5 vs. 8.4 months), with a hazard ratio favoring T-DXd. Toxicities included myelosuppression and ILD, which require careful monitoring but were generally manageable within the context of the trial [83]. Although the primary analysis of DESTINY-Gastric01 focused on patients with high-level HER2-positive disease, exploratory cohorts included patients with low-level HER2 expression (IHC 2+/ISH-negative or IHC 1+). In these exploratory subgroups, T-DXd demonstrated signals of antitumor activity, with a confirmed objective response rate (ORR ≥ 4 weeks) of 26.3% (95% CI, 9.1–51.2%) in the IHC 2+/ISH-negative cohort and 9.5% (95% CI, 1.2–30.4%) in the IHC 1+ cohort, suggesting potential clinical benefit in HER2-low GC [84].

These exploratory findings support the biological rationale for further evaluation of T-DXd in this population and have been reinforced by subsequent studies investigating ADC-based strategies in HER2-low gastric and GEJ tumors. Building on these results, DESTINY-Gastric03 is an ongoing phase I study designed to assess the safety, pharmacokinetics, and preliminary antitumor activity of T-DXd, administered as monotherapy or in combination with chemotherapy and/or immunotherapy, in patients with advanced GC/GEJ adenocarcinoma (NCT04379596). Mature efficacy data, including HER2-low–specific outcomes, are not yet available [85].

Several trials specifically focus on combination strategies involving T-DXd. A phase I study evaluating T-DXd in combination with ramucirumab aims to determine the recommended phase II dose and to assess the safety and early efficacy of this regimen in advanced GC/GEJ adenocarcinoma with HER2-low expression (NCT05894824) [79]. In the first-line setting, the EPOC2203 trial (jRCT2031230477) explores the combination of T-DXd with nivolumab and CAPOX chemotherapy in patients with metastatic GEJ adenocarcinoma, with dedicated enrollment of HER2-low disease [77,86].

Neoadjuvant approaches are also under investigation. One phase II study evaluates T-DXd as neoadjuvant monotherapy in patients with HER2-positive GC, while an additional cohort explores the combination of T-DXd with capecitabine and the PD-L1 inhibitor durvalumab, aiming to assess feasibility, safety, and preliminary efficacy in the perioperative setting (NCT05034887/EPOC2003). In this study, the monotherapy cohort includes two predefined analysis sets: a primary analysis enrolling patients with HER2 overexpression (IHC 3+ or IHC 2+/ISH-positive); and an exploratory analysis including patients with low HER2 expression (IHC 1+ or IHC 2+/ISH-negative) and elevated circulating HER2 extracellular domain levels (HER2-ECD > 11.6 ng/mL). In contrast, the combination cohort is restricted to patients with HER2-overexpressing GC/GEJ adenocarcinoma [87].

Beyond T-DXd, DV is being actively investigated in multiple clinical trials. In a multicenter, open-label phase I trial (NCT04280341), RC48 in combination with the PD-1 inhibitor toripalimab demonstrated a manageable safety profile and encouraging antitumor activity in patients with HER2-positive and low HER2-expressing gastric/GEJ cancer. Among patients with GC/GEJ cancer treated at the recommended phase II dose, DV plus toripalimab produced objective responses in both HER2-positive and HER2-low populations, with ORRs of 56% (5/9; 95% CI, 21–86) and 46% (6/13; 95% CI, 19–75), respectively [69,88].

The phase II/III multicenter trial NCT05980481 is evaluating RC48-based combination strategies as first-line treatment in patients with locally advanced or metastatic gastric or GEJ cancer across different levels of HER2 expression. The study includes experimental arms combining DV with the PD-1 inhibitor toripalimab and chemotherapy, with or without trastuzumab, as well as active comparator regimens. Patients are stratified according to trial-defined HER2 status, including HER2-high (IHC 3+ or IHC 2+/ISH+), HER2-intermediate/low (IHC 2+/ISH-negative or IHC 1+) corresponding to HER2-low as defined in this review, and HER2-zero (IHC 0) [78].

The phase I interventional trial NCT06078982 is specifically designed to evaluate the combination of the RC48-ADC with the PD-1 inhibitor toripalimab as later-line treatment in patients with advanced HER2-low expressing gastric or GEJ cancer. Investigational therapy consists of intravenous DV administered every two weeks in combination with toripalimab at defined intervals. This trial represents one of the few prospective clinical efforts to assess the safety and preliminary efficacy of ADC-based immunotherapy combinations exclusively in the HER2-low population, although no efficacy outcomes have been reported yet, as the study remains ongoing and results have not been published [89].

Another early-phase study (NCT06085755) evaluates the combination of T-DXd with the EGFR inhibitor afatinib in second- and third-line settings, with an emphasis on dose optimization and safety monitoring in HER2-low GC. This trial includes patients with low HER2 expression, aiming to explore whether dual HER2 and EGFR pathway inhibition can enhance responses in this molecularly defined subgroup. As of now, the study remains ongoing and has not reported mature efficacy results [90].

Collectively, these trials reflect a rapidly evolving clinical landscape in which HER2-low expression is increasingly recognized as a relevant therapeutic context. Although most studies are early phase and ongoing, their results are expected to clarify the role of ADC-based regimens, refine patient selection, and inform future treatment strategies for HER2-low gastric and GEJ cancer.

## 6. Safety Considerations for HER2-Directed ADCs

ADCs have introduced a distinct toxicity profile in HER2-expressing GC/GEJ that differs from conventional (unconjugated) anti-HER2 monoclonal antibodies. Consequently, safety monitoring should be proactive and structured, with particular attention to ILD/pneumonitis (notably with T-DXd), myelosuppression, gastrointestinal (GI) adverse events, and peripheral neuropathy—the latter being more typical of microtubule inhibitor–payload ADCs, such as vedotin constructs based on monomethyl auristatin E (MMAE) [91].

### 6.1. Interstitial Lung Disease/Pneumonitis

Among HER2-directed ADCs used in gastric/GEJ cancer, T-DXd is associated with a clinically significant pulmonary risk, with ILD/pneumonitis recognized as an adverse event of special interest that can be life-threatening or fatal. In DESTINY-Gastric04, adjudicated ILD/pneumonitis was reported more frequently with T-DXd than with ramucirumab–paclitaxel, although most events were low grade (Table 3) [92]. In DESTINY-Gastric02, rare fatal ILD/pneumonitis events were reported, reinforcing the need for early recognition, treatment interruption and prompt management [93]. Across prescribing information, ILD/pneumonitis is highlighted as a key safety warning with documented fatal cases, supporting proactive monitoring (baseline respiratory assessment, low threshold for CT imaging), immediate treatment interruption for suspected ILD, and prompt corticosteroids per severity, with discontinuation thresholds defined in the label [94]. In HER2-positive gastric/GEJ cancer treated with T-DXd (ENHERTU) at 6.4 mg/kg, the median time to first onset of ILD/pneumonitis is 2.8 months [94].

### 6.2. Myelosuppression

Hematologic toxicity is a frequent and clinically relevant class effect of HER2-directed ADCs in GC/GEJ and typically occurs early during therapy, supporting routine complete blood count (CBC) monitoring before each dose and timely dose modifications when clinically indicated [94]. At the 6.4 mg/kg gastric dose of T-DXd, decreased neutrophil count was reported in 72% of patients, including grade 3–4 events in 51%, with a median time to first onset of 16 days (range, 4–187); febrile neutropenia occurred in 4.8% [94]. In DESTINY-Gastric01, myelosuppression is explicitly emphasized among the key toxic effects alongside ILD. T-DXd was associated with substantial grade ≥ 3 hematologic toxicity compared with PC, most notably neutropenia, anemia, and leukopenia [83]. Beyond T-DXd, MMAE payload ADCs such as RC48 have also been associated with clinically significant cytopenias. In a phase I study enriched for GC, decreased neutrophil and white blood cell counts were among the main severe hematologic events, and febrile neutropenia-related deaths were reported (Table 3) [72]. These findings reinforce the need for CBC surveillance and infection vigilance.

### 6.3. Gastrointestinal Adverse Events

GI toxicity is among the most frequent tolerability issues with HER2-directed ADCs in GC/GEJ, typically presenting as nausea, vomiting, diarrhea, constipation, and appetite loss, and may necessitate proactive antiemetic prophylaxis, hydration strategies, and early diarrhea management to maintain dose intensity. Across DESTINY-Gastric01 and DESTINY-Gastric02, T-DXd was consistently associated with frequent GI toxicity—particularly nausea, vomiting, diarrhea, and decreased appetite (Table 3)—supporting proactive antiemetic prophylaxis and early supportive care [83,93]. Beyond T-DXd, GI adverse events were also common with the MMAE-based ADC RC48 in the phase I RC48 study [72].

### 6.4. Peripheral Neuropathy (Payload-Dependent)

Neurotoxicity associated with HER2-directed ADCs is largely payload-driven and is more commonly observed with microtubule inhibitor–based constructs (e.g., MMAE-containing agents) than with topoisomerase I–payload ADCs such as T-DXd. In a phase II GC/GEJ adenocarcinoma cohort (n = 125), hypoesthesia occurred in 32.8% of patients with grade 3–5 events in 3.2%. In the phase I RC48 study, hypoesthesia was among the most frequent grade ≥ 3 treatment-related adverse events (14.0%), supporting the view that neuropathy represents a dose- and exposure-sensitive tolerability issue for MMAE-based ADCs [71,72]. Taken together, these data support routine neuropathy screening at each visit, attention to potential cumulative effects, and timely protocol-guided dose interruption and/or reduction when sensory neuropathy develops or worsens.

### 6.5. Left Ventricular Dysfunction

Although uncommon, left ventricular ejection fraction (LVEF) decline has been reported with T-DXd across tumor types (4.6% at 5.4 mg/kg; grade 3–4, 0.6%), supporting baseline and periodic LVEF monitoring. In GC/GEJ cohorts treated at 6.4 mg/kg, no clinical heart failure events were reported, but asymptomatic grade 2 LVEF decreases have been detected—8% with echocardiography per prescribing information and 10.5% (6 patients) with on-study LVEF assessments in DESTINY-Gastric02 [93,94].

## 7. Strengths, Limitations and Future Directions

Our review has several strengths. It integrates pathology-oriented aspects of HER2 assessment with the emerging therapeutic evidence for HER2-directed ADCs in GC/GEJ adenocarcinoma, while also incorporating consensus recommendations, registered clinical trials, safety considerations, and cross-tumor perspectives. This integrated approach may help clarify practical challenges in interpreting HER2-low status and support future biomarker-driven trial design.

This review was designed as a narrative synthesis rather than a systematic review; therefore, we did not apply a PRISMA-style selection workflow, formal risk-of-bias tools, or quantitative meta-analysis. In addition, the clinical evidence base for HER2-low GC/GEJ is still developing and is largely derived from early-phase studies and exploratory analyses, often with heterogeneous populations and endpoints. Finally, although HER2-low is defined as IHC 1+ or IHC 2+/ISH-negative, differences in sampling (biopsy vs. resection; primary vs. metastatic site) and interpretative challenges in distinguishing IHC 0 vs. 1+ can affect cross-study comparability.

HER2-low-specific differences according to anatomical subsite, including Siewert type I versus type II GEJ adenocarcinomas, remain insufficiently defined and should be addressed in future biomarker studies.

Another important future direction is the characterization of resistance mechanisms to HER2-directed ADCs in HER2-low GC/GEJ. Resistance to HER2-targeted therapy in gastroesophageal adenocarcinoma may be intrinsic (primary resistance) or acquired upon treatment (secondary resistance) and is driven by multiple mechanisms [95]. Reported mechanisms include loss or reduction in HER2 expression; receptor alterations that impair antibody binding; masking of the HER2 binding domain—for example, by mucins such as MUC1—activation of alternative receptor pathways such as HER3 or MET (mesenchymal–epithelial transition factor); and downstream activation of phosphoinositide 3-kinase/protein kinase B (PI3K/AKT) or mitogen-activated protein kinase (MAPK) signaling [96,97,98,99]. In HER2-low disease, low baseline antigen density and spatial heterogeneity may be particularly relevant because ADC efficacy depends on sufficient target engagement, internalization, and intracellular payload release. In addition, ADC-specific resistance may involve impaired receptor internalization, altered endosomal–lysosomal trafficking, reduced intracellular payload release, drug efflux, and payload-specific resistance mechanisms [100,101]. Although several resistance mechanisms are biologically plausible and supported by preclinical evidence, their frequency and clinical impact remain insufficiently defined in GC/GEJ, especially in HER2-low disease. Further identification of resistance mechanisms in clinical samples, together with strategies designed to target or bypass these pathways, may improve patient selection, treatment sequencing, and outcomes with HER2-directed ADCs. A challenging subject refers to the particular histological types such mixed adenoneuroendocrine carcinomas [102,103], primary choriocarcinomas [104] or carcinomas with epithelial–mesenchymal transitions [105]. No guidelines for HER-2 interpretation in such cases are available.

Future research should prioritize prospective trials dedicated to HER2-low GC/GEJ with clearly prespecified HER2-low definitions and adequately powered peer-reviewed efficacy endpoints. Additional priorities include biomarker strategies beyond binary IHC thresholds, resistance characterization, and rational treatment sequencing. Combination approaches involving immunotherapy, chemotherapy, and anti-angiogenic agents should also be evaluated in clearly defined clinical settings.

## 8. Conclusions

HER2-low GC/GEJ adenocarcinoma occupies a clinically important but still unsettled space between conventional HER2-negative disease and established HER2-positive therapeutic algorithms. Low-level HER2 expression provides a biologically plausible entry point for HER2-directed ADC strategies, but current evidence is not yet sufficient to guide routine treatment selection outside approved indications or clinical trials. Future progress will require harmonized pathology workflows, improved characterization of spatial and temporal HER2 heterogeneity, and biomarker approaches that move beyond binary IHC categories. Prospective studies should clarify which patients derive durable benefit from ADC-based regimens and how these therapies should be combined, sequenced, and adapted after resistance.

## Figures and Tables

**Table 1:** Evidence levels for HER2-directed strategies in GC/GEJ adenocarcinoma.

Evidence Category	Subcategory	Examples	Practical Interpretation
Established	HER2-positive disease	ToGA trial; guideline-based trastuzumab + chemotherapy for HER2-positive advanced GC/GEJ adenocarcinoma	HER2-directed therapy is established only for HER2-positive disease, within approved indications and guideline-based treatment algorithms.
Investigational	Exploratory subgroup signals	DESTINY-Gastric01 HER2-low cohorts	Exploratory HER2-low cohorts suggested T-DXd activity, especially in IHC 2+/ISH-negative disease, but require prospective validation.
	Early-phase ADC-based combinations	RC48/DV-based combinations	RC48/DV plus toripalimab showed manageable safety profile and early efficacy signals, but remains investigational.
	Dedicated ongoing HER2-low trials	T-DXd + ramucirumab (NCT05894824); T-DXd + nivolumab + CAPOX/EPOC2203 (jRCT2031230477);	These studies prospectively test HER2-low-directed strategies, but mature efficacy and safety data are not yet available.

Abbreviations: ADC, antibody–drug conjugate; CAPOX, capecitabine plus oxaliplatin; RC48/DV, RC48/disitamab vedotin; T-DXd, trastuzumab deruxtecan. Note: HER2-low strategies are summarized according to evidence maturity and should not be interpreted as established routine treatment indications in GC/GEJ adenocarcinoma.

**Table 2:** Overview of selected clinical trials in HER2-low gastric and GEJ cancer.

Trial (Identifier)	Phase	HER2-Low Focus	Therapeutic Agent	Line of Therapy	Enrollment
DESTINY-Gastric01 (NCT03329690)	II	Exploratory subgroup	T-DXd + PC (irinotecan/ paclitaxel monotherapy)	≥2 L	233 (actual)
DESTINY-Gastric03 (NCT04379596)	Ib/II	Exploratory subgroup	T-DXd ± chemo/immunotherapy	Multiple lines	413 (estimated)
NCT05894824	Ib/II	Dedicated cohort	T-DXd + ramucirumab	2 L	58 (estimated)
jRCT2031230477 (EPOC2203)	Ib/II	Dedicated cohort	T-DXd + nivolumab and CAPOX	1 L	31 (planned)
NCT05034887 (EPOC2003)	II	Exploratory subgroup	T-DXd monotherapy vs. T-DXd + durvalumab + capecitabine	Neoadjuvant	64 (estimated)
NCT04280341	I	Exploratory subgroup	RC48 + anti-PD-1	Multiple lines	50 (estimated)
NCT05980481	II/III	Exploratory subgroup	RC48 + toripalimab + chemo/trastuzumab	1 L	201 (estimated)
NCT06078982	I	Dedicated cohort	RC48 + toripalimab	≥2 L	39 (estimated)
NCT06085755	I/II	Dedicated cohort	T-DXd + afatinib	≥2 L	61 (estimated)

Notes: HER2-low was defined as IHC 1+ or IHC 2+ with negative ISH. Enrollment reflects actual, planned, or estimated sample sizes as reported in trial registries. Abbreviations: GEJ, gastroesophageal junction; PC, physician’s choice; T-DXd, trastuzumab deruxtecan; RC48, disitamab vedotin; PD-1, programmed cell death protein 1; 1 L, first-line; 2 L, second-line; ≥2 L, second- or later lines of therapy. Trial identifiers and enrollment were obtained from trial registries (e.g., ClinicalTrials.gov and regional registries) and corresponding publications are cited in the text.

**Table 3:** Comparative summary of selected major adverse events reported with HER2-directed ADC in GC/GEJ adenocarcinoma.

Toxicity Domain	Study	Therapeutic Agent	Adverse Events	Any Grade	Grade ≥ 3
Pulmonary Toxicity	DESTINY-Gastric04 [92]	T-DXd	ILD/pneumonitis	13.9%	Grade 3: one patient
		Ramucirumab-paclitaxel		1.3%	Grade 3: 1.3% Grade 5: one patient
	DESTINY-Gastric02 [93]	T-DXd	ILD/pneumonitis	10.1%	Grade 5: 2.5%
Hematologic Toxicity	DESTINY-Gastric01/DESTINY-Gastric02 [83,93]	T-DXd	Decreased NC	63%/8.9%	51%/7.6%
			Anemia	58%/24.1%	38%/13.9%
			Leukopenia	38%/5.1%	21%/6.3%
	Phase I study of MMAE conjugate RC48-ADC [72]	RC48	Decreased NC	56.1%	19.3% and two deaths
			WBC decreased	64.9%	17.5%
Gastrointestinal Toxicity	DESTINY-Gastric01/ DESTINY-Gastric02 [83,93]	T-DXd	Nausea	63%/59.5%	5%/7.6%
			Vomiting	26%/41.8%	NR/2.5%
			Diarrhea	32%/35.4%	2%/1.3%
	Phase I study of MMAE conjugate RC48-ADC [72]	RC48	Nausea/vomiting	33.3%/19.3%	Vomiting: one patient
			Diarrhea	24.6%	
Neurologic Toxicity	Phase I study of MMAE conjugate RC48-ADC [72]	RC48	Hypoesthesia/ fatigue	43.9%/56.1%	14.0%/5.3%
	A single-arm phase II study of RC48 [71]	RC48	Asthenia/ hypoesthesia	53.6%/32.8%	2.4%/3.2%

Notes: For rows combining DESTINY-Gastric01 and DESTINY-Gastric02, values are presented in the same order: DESTINY-Gastric01/DESTINY-Gastric02. When two adverse events are listed in the same cell, the corresponding percentages are presented in the same order as the adverse events. Percentages were calculated from reported event counts when denominators were clearly provided. This table summarizes selected major adverse events and is not intended as an exhaustive safety listing. Abbreviations: ADC, antibody–drug conjugate; HER2, human epidermal growth factor receptor 2; ILD, interstitial lung disease; MMAE, monomethyl auristatin E; NC, neutrophil count; NR, not reported; RC48, disitamab vedotin; T-DXd, trastuzumab deruxtecan; WBC, white blood cell.

## Data Availability

The original contributions presented in this study are included in the article/Supplementary Material. Further inquiries can be directed to the corresponding author.

## Supplementary Materials

The following supporting information can be downloaded at: https://www.mdpi.com/article/10.3390/ijms27114673/s1.

## Abbreviations

The following abbreviations are used in this manuscript:

ACRG	Asian Cancer Research Group
ADC/ADCs	Antibody–drug conjugate(s)
ASCO	American Society of Clinical Oncology
CAP	College of American Pathologists
CAPOX	Capecitabine plus oxaliplatin
CBC	Complete blood count
CI	Confidence interval
CIN	Chromosomal instability
DCR	Disease control rate
EBV	Epstein–Barr virus
EGFR	Epidermal growth factor receptor
ERBB2	ERBB2 gene (HER2 gene)
ESMO	European Society for Medical Oncology
FISH	Fluorescence in situ hybridization
GC	Gastric cancer
GEJ	Gastroesophageal junction
GI	Gastrointestinal
GS	Genomically stable
HER2	Human epidermal growth factor receptor 2
HR	Hazard ratio
IHC	Immunohistochemistry
ILD	Interstitial lung disease
ISH	In situ hybridization
LVEF	Left ventricular ejection fraction
MMAE	Monomethyl auristatin E
MSI	Microsatellite instability
MSS/EMT	Microsatellite stable/epithelial–mesenchymal transition
MSS/TP53−	Microsatellite stable/TP53 inactive
MSS/TP53+	Microsatellite stable/TP53 aberrant/active
NSCLC	Non-small cell lung cancer
ORR	Objective response rate
OS	Overall survival
PC	Physician’s choice
PD-1	Programmed cell death protein 1
PDAC	Pancreatic ductal adenocarcinoma
PD-L1	Programmed death-ligand 1
PFS	Progression-free survival
RC48/DV	Disitamab vedotin
TCGA	The Cancer Genome Atlas
T-DXd	Trastuzumab deruxtecan
TP53	Tumor protein p53 gene
UC	Urothelial carcinoma
UCS	Uterine carcinosarcoma
